# Patients Infected with CRF07_BC Have Significantly Lower Viral Loads than Patients with HIV-1 Subtype B: Mechanism and Impact on Disease Progression

**DOI:** 10.1371/journal.pone.0114441

**Published:** 2014-12-11

**Authors:** Szu-Wei Huang, Sheng-Fan Wang, Yu-Ting Lin, Chia-Hung Yen, Chih-Hao Lee, Wing-Wai Wong, Hung-Chin Tsai, Chia-Jui Yang, Bor-Shen Hu, Yu-Huei Lin, Chin-Tien Wang, Jaang-Jiun Wang, Zixin Hu, Daniel R. Kuritzkes, Yen-Hsu Chen, Yi-Ming Arthur Chen

**Affiliations:** 1 Center for Infectious Disease and Cancer Research (CICAR), Kaohsiung Medical University, Kaohsiung, Taiwan; 2 Institute of Microbiology and Immunology, National Yang-Ming University, Taipei, Taiwan; 3 Department of Medical Laboratory Science and Biotechnology, Kaohsiung Medical University, Kaohsiung, Taiwan; 4 Section of Infectious Diseases, Department of Internal Medicine, Taipei Veterans' General Hospital, Taipei, Taiwan; 5 Section of Infectious Diseases, Department of Internal Medicine, Kaohsiung Veterans' General Hospital, Kaohsiung, Taiwan; 6 Section of Infectious Diseases, Department of Internal Medicine, Far Eastern Memorial Hospital, New Taipei City, Taiwan; 7 Section of Infectious Diseases, Department of Internal Medicine, Taipei City Hospital, Taipei, Taiwan; 8 Section of Infectious Diseases, Department of Internal Medicine, Taichung Veterans' General Hospital, Taichung, Taiwan; 9 Institute of Clinical Medicine, National Yang-Ming University, Taipei, Taiwan; 10 Department of Pediatrics, Children's Healthcare of Atlanta and Emory University School of Medicine, Atlanta, Georgia, United States of America; 11 Division of Infectious Diseases, Brigham and Women's Hospital and Harvard Medical School, Boston, Massachusetts, United States of America; 12 School of Medicine, Graduate Institute of Medicine, Sepsis Research Center, College of Medicine, Kaohsiung Medical University, Kaohsiung, Taiwan; 13 Division of Infectious Diseases, Department of Internal Medicine, Kaohsiung Medical University Hospital, Kaohsiung, Taiwan; 14 Department of Microbiology, Institute of Medical Research and Institute of Clinical Medicine, College of Medicine, Kaohsiung Medical University, Kaohsiung, Taiwan; Institute of Infection and Global Health, United Kingdom

## Abstract

The circulating recombinant form (CRF) 07_BC is the most prevalent HIV-1 strain among injection drug users (IDUs) in Taiwan. It contains a 7 amino-acid deletion in its p6^gag^. We conducted a cohort study to compare viral loads and CD4 cell count changes between patients infected with subtype B and CRF07_BC and to elucidate its mechanism. Twenty-one patients infected with CRF07_BC and 59 patients with subtype B were selected from a cohort of 667 HIV-1/AIDS patients whom have been followed up for 3 years. Generalized estimated equation was used to analyze their clinical data and the results showed that patients infected with CRF07_BC had significantly lower viral loads (about 58,000 copies per ml less) than patients with subtype B infection (*p* = 0.002). The replicative capacity of nine CRF07_BC and four subtype B isolates were compared and the results showed that the former had significantly lower replicative capacity than the latter although all of them were CCR5- tropic and non-syncytium inducing viruses. An HIV-1-NL4-3 mutant virus which contains a 7 amino-acid deletion in p6^gag^ (designated as 7d virus) was generated and its live cycle was investigated. The results showed that 7d virus had significantly lower replication capacity, poorer protease-mediated processing and viral proteins production. Electron microscopic examination of cells infected with wild-type or 7d virus demonstrated that the 7d virus had poorer and slower viral maturation processes: more viruses attached to the cell membrane and higher proportion of immature virions outside the cells. The interaction between p6^gag^ and Alix protein was less efficient in cells infected with 7d virus. In conclusion, patients infected with CRF07_BC had significantly lower viral loads than patients infected with subtype B and it may due to the deletion of 7 amino acids which overlaps with Alix protein-binding domain of the p6^gag^.

## Introduction

Understanding the factors affecting AIDS disease progression is very important for clinical management and counseling. It has been reported that HIV-1 patients infected with different subtypes have different rates of disease progression [1]–[3]. In Kenya, patients infected with subtype D recombinant virus had significantly faster disease progression than patients infected with subtype A in spite that they had similar viral loads [2]. A meta-analysis indicated that the trend of disease progression among different HIV-1 subtype in a descending order was subtype C>D>AE>G>A [4].

By the end of 2013, 27,366 individuals (including 891 foreigners) were reported as infected with HIV-1 by the Taiwan's Centers for Disease Control (CDC). Risk factor analyses showed that more than 50% of the HIV-1/AIDS patients were men who have sex with men (MSM) and about 25% were injection drug users (IDUs) [5]. In terms of subtype distribution, subtype B, CRF01_AE and CRF07_BC were predominant in MSM, heterosexuals, and IDUs respectively [6]–[10]. There are an estimated 60,000 to 100,000 IDUs in Taiwan and about 15% of them are infected with HIV-1 [9]. Since 83% of those HIV-1-infected IDUs may be infected with CRF07_BC [10], the numbers of IDUs in Taiwan who may be infected with CRF07_BC are between 7,470–12,450. Therefore, it is very important to understand the natural history and disease progression of CRF07_BC infection.

Previously, our full-length sequencing results indicated that all the Taiwanese CRF07_BC strains contain a signature 7 amino-acid deletion in p6^gag^ [8]. The p6^gag^ contains two motifs- PTAP and YPX_n_L (X can vary in sequence) which are important for viral assembling and budding. YPX_n_L motif is located between amino acid residues 36 and 44 at its C-terminal region and it interacts with AIP1 (apoptosis-linked gene 2-interacting protein, also known as Alix) [11], [12]. Since a 7 amino-acid deletion signature is overlapping with this motif, we hypothesized that such deletion may affect the viral life cycle, especially during virus assembly. In this study, 21 patients infected with CRF07_BC and 59 patients with subtype B were selected from a cohort of 667 HIV-1/AIDS patients whom have been followed up for more than 3 years. A GEE model was used to analyze multiple time points data and demonstrated that patients with CRF07_BC infection had significantly lower viral loads than patients with subtype B infection and it was mainly associated with the viral subtypes. Subsequently, we used both clinical isolates and molecular clones with specific deletion of those 7 amino-acid from p6^gag^ to elucidate the mechanism and the results indicated that the lower replication capacity, poorer protease-mediated processing and viral proteins production of CRF07_BC were due to a 7 amino-acid deletion in its p6^gag^ domain.

## Methods

### Patient Cohort and Study Design

In 2010, we established Taiwan HIV-1 Observational Database (TwHOD) to study the natural history and clinical aspects of Taiwanese HIV-1/AIDS patients. The TwHOD is a collaborative cohort study that involves the following hospitals located in the northern region (Taipei Veterans' General Hospital, Far Eastern Memorial Hospital, and Taipei City Hospital), central region (Taichung Veterans' General Hospital) and southern region (Kaohsiung Veterans' General Hospital) of Taiwan. The study was approved by the Institutional Review Boards (IRB) of all the participating hospitals. Written informed consent was obtained from patients who agreed to participate in this study. The procedure of data collection was similar to that reported by TREAT Asia HIV-1 Observational Database [13]. By the end of 2012, 667 HIV-1/AIDS patients enrolled in this study and the HIV-1 subtypes of 272 patients were determined. For the nested case control study, we selected 21 male treatment naïve patients infected with CRF07_BC and matched them with 59 subtype B-infected patients by gender, age and risk factor. Eleven patients who were infected with CRF07_BC were excluded due to gender (3 were female patients), previous antiretroviral therapy (2 patients) or lack of CD4 cell count or viral loads data (6 patients).

### Subtyping and Isolation of HIV-1 viruses

HIV-1 subtypes were determined as described previously [14]. Nine CRF07_BC and 4 subtype B strains from treatment-naïve IDUs were obtained by using standard peripheral blood mononuclear cells (PBMCs) co-culture methods [15]. We enrolled treatment naïve HIV-1-infected IDU patients with CD4 cell counts of more than 500 cells/mm^3^. Initially, we selected 10 treatment naïve patients with CRF07_BC and 5 patients with subtype B infection for the virus isolation experiments. Eventually, 9 CRF07_BC and 4 subtype B isolates were obtained.

### Determination of Co-receptor Usage and Syncytium Inducing (SI) Ability

Chemokine co-receptor usage and the SI ability were determined as described previously [16], [17]. In terms of sequence analysis of V3 region of env gene, co-receptor usage was determined as described previously [18]–[20].

### Generation of Recombinant Viruses

To generate HIV-1 infectious recombinant viruses with or without a 7 amino-acid deletion at its p6^gag^, MT2 cells were co-transfected with a linear marker plasmid pNL43HIVΔ*PR.RT*BstEII*nef*-*GFP* and one of two linear plasmids pGEM-NCRT or pGEM-NCRT-7d using an electroporation method [21]. Briefly, a 1,652-bp fragment encompassing the coding regions of HIV-1 nucleocapsid protein-p6-protease-reverse transcriptase was amplified from plasmid pNL4-3 (corresponding to nucleotides 1827 to 3649 of the HIV-1 NL4-3 sequence) using PCR. It was sub-cloned into pGEM-T vector (Promega, Madison, Wisconsin) to generate a plasmid designated as pGEM-NCRT. Subsequently, we used PCR-based site-directed mutagenesis to delete nucleotide sequences 2220–2240 of HIV-1 NL4-3 to generate a plasmid containing a 7 amino-acid deletion in its p6^gag^ (pGEM-NCRT-7d).

### Growth Kinetic Assay

Growth kinetic of primary isolates and infectious recombinant viruses were measured in PBMCs and MT2 cells, respectively and described with some modifications [17]. Cells were plated in 24-well plates at 10^6^ cells/well in 1 ml of RPMI 1640 medium, and 3,000 50% tissue culture infective dose (TCID_50_) of HIV-1 viruses were added. The cultures were split every 3-4 days by replacing 50% of the culture with the same volume of fresh medium and p24 quantified as a measure of ongoing virus replication. Growth kinetic of infectious recombinant viruses was measured in MT2 cells, as described elsewhere [17] with modification. A total of 2×10^6^ cells were infected with 2,000 TCID_50_ of viruses. After incubation for 2 hours at 37°C, cells were washed twice with phosphate-buffered saline (PBS) and resuspended in RPMI 1640 medium. Triplicate cultures were tested, and viral growth was determined by HIV-1 p24 levels on days 2, 4, 6, 8, 10, 12 and 14. HIV-1 p24 antigen determined by enzyme-linked immunosorbent assay (ELISA) (PerkinElmer, Waltham, USA) was considered an indicator of virus replication.

### Western Blot (WB) Assay

The details of WB have been described previously [22]. HIV-1 Gag proteins were detected by anti-p24^gag^ mouse monoclonal antibody (clone 183-H12-5C) [23]. RT was detected by anti-RT mouse monoclonal antibody [24]. Protease was detected by anti-HIV protease mouse monoclonal antibody (Abcam). The bound antibody was detected by horseradish peroxidase-conjugated anti-mouse immunoglobulin secondary antibody (Amersham Corp.). Image J software (version 1.47) was used to analyze the intensity of reactive bands in WB.

### Electron microscopy

Infected MAGIC-5 cells [25] were fixed in 2.5% glutaraldehyde-0.2M sodium cacodylate solution overnight at 4°C, and then fixed with 1% OsO_4_ in PBS for 1.5 hours. Specimens were then dehydrated in graded ethanol solution and embedded in Epon. Ultrathin sections were stained with uranyl acetate and lead citrate, and images were obtained using Jeol JEM-2000EXII transmission electron microscope (TEM).

### Indirect Immunofluorescent Antibody (IFA) Staining and Total Internal Reflection Fluorescence (TIRF)

To detect and quantify the interaction between p6^gag^ and Alix, IFA staining with TIRF and super-resolution fluorescence localization imaging methods were used (Leica SR GSD) [26], [27] for immunostaining, Gag was detected by anti-p24^Gag^ mouse monoclonal antibody. Alix was detected by anti-Alix rabbit polyclonal antibody. The secondary antibodies were anti-mouse and anti-rabbit fluorescence (Alexa 488 and Alexa 647)-conjugated antibodies. Phosphate buffered saline containing 100 mM β-mercaptoethylamine (MEA) was used for SR fluorescence localization imaging. Imaging fields were magnified using a 100× oil objective (Leica) with a 1.47 numerical aperture and 1.6× optical magnification. The penetration depth of the excitation laser source for TIRF and super-resolution imaging was 200 nm. TIRF fluorescence image stacks consisting of over 30,000 frames were used to calculate SR fluorescence images. A two-dimensional spatial histogram map in each fluorescence channel was calculated using the SR images with an effective pixel size of 20 nm. The co-localization coefficients of two proteins were quantified by a combination of Manders analysis and two-dimensional spatial histogram maps of two fluorescence channels, with the fluorescence background removed during intensity-based co-localization analysis.

### Statistical Analysis

A multivariate linear generalized estimating equations (GEE) model was performed to identify factors associated with the changes of CD4 cell count or viral loads. One-way ANOVA and Tukey's post hoc test were used to compare the p24 antigen levels between different subtypes or infectious recombinant viruses. SAS statistic software (SAS version 9.1; SAS Institute, Cary, North Carolina, USA) was used with significance level set at p<0.05.

## Results

### Study Population Characteristics

In total, 667 HIV-1/AIDS patients were recruited in this study. The median follow-up period was 46 months. In terms of socio-demographic variables, the age of the participants ranged from 15 to 81 years at diagnosis of HIV-1 infection. The median age was 31 years and 94.0% were men. The majority of the patients were MSM (388/600 [64.7%]) and 22.5% were IDUs. The results of HBsAg and HCV antibody tests were available for 301 (45.1%) and 388 (58.2%) patients, respectively. Seventy-one (23.6%) patients were HBsAg positive and 121 patients (31.2%) had anti-HCV antibodies. There were 466 patients (69.9%) under ART (Table 1).

**Table 1:** Demographic data, risk factors and clinical characteristics of HIV-1-infected patients recruited in the study.

	HIV-1 infected population					
	Male (%)		Female (%)		Total (%)	
	(N = 627)		(N = 40)		(N = 667)	
**Age (yrs)**	n = 612		n = 37		n = 649	
15–29	287	(46.9)	12	(32.4)	299	(49.2)
30–50	283	(46.2)	19	(51.4)	302	(42.5)
≥50	42	(6.9)	6	(16.2)	48	(8.3)
**Mode of infection**	n = 561		n = 39		n = 600	
Homosexual contact	388	(69.2)	0	(0)	388	(64.7)
Heterosexual contact	51	(9.1)	20	(51.3)	71	(11.8)
Injecting drug use	116	(20.7)	19	(48.7)	135	(22.5)
Receipt of blood products	6	(1.1)	0	(0)	6	(1.0)
**HBV coinfection**	n = 280		n = 21		n = 301	
HBsAg, positive	68	(24.3)	3	(14.3)	71	(23.6)
**HCV coinfection**	n = 361		n = 27		n = 388	
HCVAb, positive	105	(29.1)	16	(59.3)	121	(31.2)
**Antiretroviral treatment**	n = 627		n = 40		n = 667	
HAART	444	(70.8)	22	(55.0)	466	(69.9)
**HIV-1 subtype**	n = 262		n = 10		n = 272	
B	215	(82.1)	4	(40.0)	219	(80.5)
C	3	(1.1)	0	(0)	3	(1.1)
CRF01_AE	14	(5.3)	3	(30.0)	17	(6.3)
CRF07_BC	29	(11.1)	3	(30.0)	32	(11.8)
CRF08_BC	1	(0.4)	0	(0)	1	(0.4)

Among 272 patients whose subtypes have been determined using nested multiplex PCR, 80.5% were subtype B, 11.8% CRF07_BC, and 6.3% CRF01_AE. Three (1.1%) patients infected with subtype C and one patient with CRF08_BC (0.4%) (Table 1). Subtype B was predominant in MSM (93.9%) and heterosexuals (69.9%). 74.3% of IDUs were infected with CRF07_BC. Notably, two MSMs were infected with CRF07_BC and they denied using intravenous drugs before. In addition, we found that there was one MSM infected with CRF08_BC and three MSM infected with subtype C (Table 2).

**Table 2:** Distribution of HIV-1 subtypes and circulating recombinant forms (CRFs) in different groups of patients recruited in this cohort study.

	Male					
Subtype/CRF	Heterosexual	MSM	IDUs	Others	Female^a^	Total
B	16 (69.6)	154 (93.9)	9 (25.7)	36 (90.0)	4 (40)	219 (80.5)
C	0 (0)	3 (1.8)	0 (0)	0 (0)	0 (0)	3 (1.1)
CRF01_AE	7 (30.4)	4 (2.4)	0 (0)	3 (7.5)	3 (30)	17 (6.3)
CRF07_BC	0 (0)	2 (1.2)	26 (74.3)	1 (2.5)	3 (30)	32 (11.8)
CRF08_BC	0 (0)	1 (0.6)	0 (0)	0 (0)	0 (0)	1 (0.4)
Total	23 (100)	164 (100)	35 (100)	40 (100)	10 (100)	272 (100)

a Includes 4 heterosexual female infected with subtype B, 3 heterosexual female infected with CRF01_AE, and 3 IDUs infected with CRF07_BC.

### Factors Affecting Disease Progression

**Figure 1:**
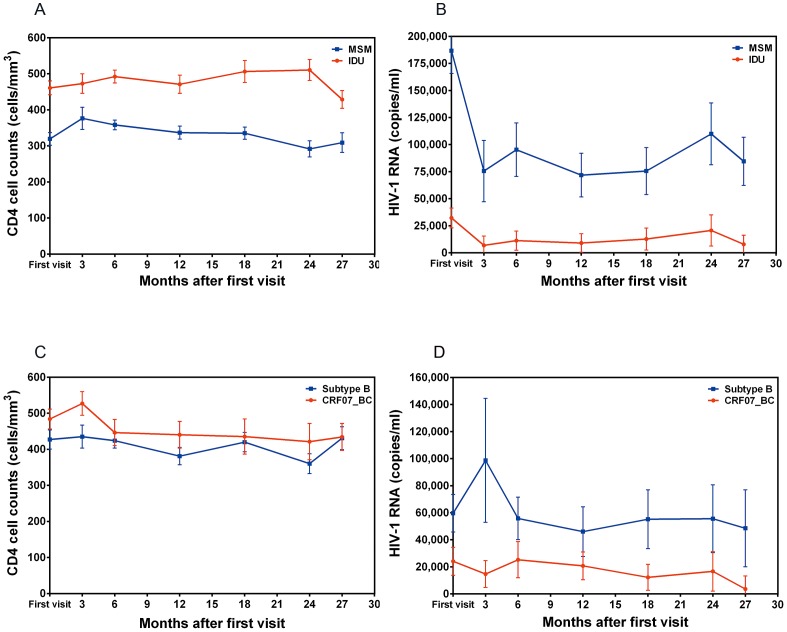
Comparisons of the changes of CD4 cell count and HIV-1 viral loads after the first clinical visit among the following four groups of treatment naïve patients. Men who have sex with men (MSM, 357 patients for CD4 cell count and 371 patients for viral loads analysis) vs. injection drug users (IDUs, 129 patients for CD4 cell count and 128 patients for viral loads analysis) (Fig. 1A and 1B); patients infected with CRF07_BC vs. infected with subtype B (Figs. 1C and 1D). A generalized estimating equation model was used for the analyses.

As shown in Figs. 1A and 1B, compared with MSM, IDUs had consistently higher CD4 cell count and lower HIV-1 viral loads over 2.5 years. A GEE model was used to identify factors associated with changes of CD4 cell count or viral loads. The variables which potentially may influence the CD4 cell count or HIV-1 viral loads were considered in this analysis. As shown in Table 3, regardless of whether “mode of infection” was added in the model (multivariate model I vs. model II), factors significantly associated with CD4 cell count changes included older age, subtypes (CRF01_AE and CRF08_BC), higher viral loads and no previous HAART. In contrast, when we analyzed factors associated with changes of viral loads, once we deleted “mode of infection” from the model (multivariate model II), we found that CRF07_BC infection became significantly associated with lower viral loads compared to subtype B infection (Table 4).

**Table 3:** Univariate and multivariate generalized estimating equations models of factors associated with CD4 cell counts.

		Univariate		Multivariate model I		Multivariate model II^a^	
	No.	difference	*p*	difference	*p*	difference	*p*
**Gender**							
Male	578						
Female	37	20.8	0.541	43.0	0.187	52.2	0.082
**Age (yrs)**							
15–29	271						
30–50	281	−31.1	0.084	−43.6	0.012	−36.4	0.040
≥50	46	−153.2	<0.001	−140.6	<0.001	−170.7	<0.001
**Mode of transmission**							
Homosexual contact	357						
Heterosexual contact	70	−59.8	0.032	−33.9	0.228	-	-
Injecting drug use	129	40.7	0.066	103.3	0.001	-	-
Receipt of blood products	6	−164.4	0.115	−118.5	0.222	-	-
**HIV-1 subtype**							
B	201						
C	3	85.6	0.317	68.2	0.384	70.5	0.367
CRF01_AE	15	−147.7	0.004	−103.3	0.041	−118.0	0.020
CRF07_BC	31	9.9	0.746	−24.4	0.510	53.1	0.084
CRF08_BC	1	−225.6	<0.001	−132.3	<0.001	−135.5	<0.001
**HIV viral loads (copies/ml)**							
<40	225						
40–400	34	−42.8	0.008	−44.1	0.007	−41.4	0.010
401–10,000	93	−107.1	<0.001	−99.0	<0.001	−97.7	<0.001
>10,000	109	−169.9	<0.001	−151.2	<0.001	−153.6	<0.001
**Received ART**							
No	261						
Yes	354	118.3	<0.001	50.0	0.005	41.6	0.020

a The mode of transmission was removed from multivariate model II.

**Table 4:** Univariate and multivariate generalized estimating equations model of factors associated with HIV-1 viral loads.

		Univariate		Multivariate model I		Multivariate model II^a^	
	No.	difference	*p*	difference	*p*	difference	*P*
**Gender**							
Male	603						
Female	38	−26767.8	<0.001	−20826.2	0.013	−26658.9	<0.001
**Age (yrs)**							
<30	286						
30–50	290	−4936.2	0.449	1520.8	0.823	−3313.5	0.633
≥50	48	−4747.7	0.627	2665.1	0.802	8246.7	0.404
**Mode of transmission**							
Homosexual contact	371						
Heterosexual contact	70	−3605.2	0.706	10465.9	0.387	-	-
Injecting drug use	128	−22329.5	<0.001	−73432.1	<0.001	-	-
Receipt of blood products	6	−7205.5	0.752	−35456.4	0.227	-	-
**HIV-1 subtype**							
B	217						
C	3	1607.7	0.940	23058.5	0.269	19596.9	0.325
CRF01_AE	16	−10620.5	0.291	−43785.9	0.004	−37612.3	0.004
CRF07_BC	30	−21153.8	0.005	−9074.4	0.472	−58045.6	<0.001
CRF08_BC	1	−7883.3	0.086	−113409.7	<0.001	−106777.7	<0.001
**CD4 cell count (cells/mm^3^)**							
≤50	22						
51–250	82	−263241.1	<0.001	−240696.2	<0.001	−246030.4	<0.001
251–500	209	−313911.1	<0.001	−290801.3	<0.001	−296881.6	<0.001
>500	160	−334294.9	<0.001	−300993.4	<0.001	−309618.0	<0.001
**Received ART**							
No	270						
Yes	371	−97661.2	<0.001	−102574.2	<0.001	−88088.5	<0.001

a The mode of transmission was removed from multivariate model II.

To evaluate the effect of HIV-1 subtypes on CD4 cell count and HIV-1 viral loads, a nested case control study which consisted of 21 patients with CRF07_BC infection and 59 patients with subtype B infection was established. They were all treatment-naïve patients and matched by age and gender. The results showed that there was no significant difference in CD4 cell count (GEE model, *p* = 0.168) (Fig. 1C). In contrast, patients infected with CRF07_BC had significantly lower viral loads than patients infected with subtype B (GEE model, *p* = 0.002) (Fig. 1D).

### CRF07_BC Isolates had Lower Replication Capacity than the Subtype B Isolates

**Figure 2:**
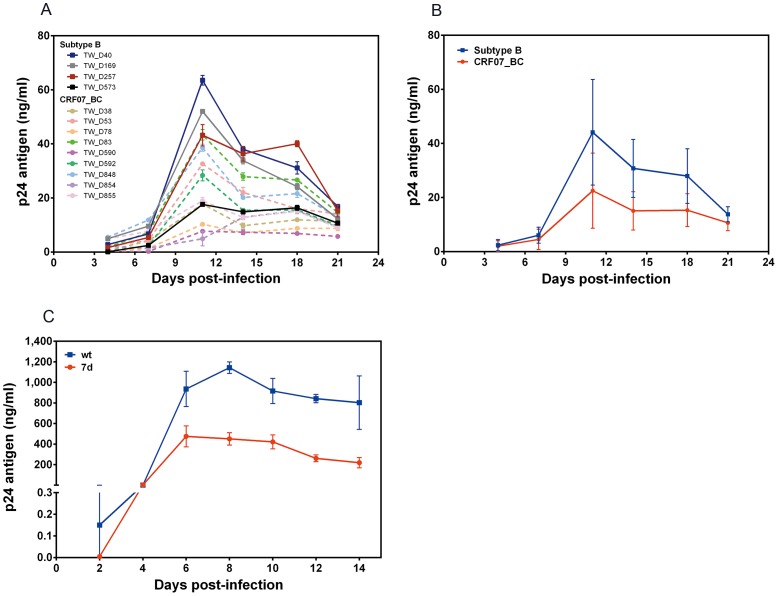
Comparison of the replication kinetics of different HIV-1 isolates from patients infected with CRF07_BC or subtype B (A and B) as well as recombinant HIV-1 virus with or without a 7 amino-acid deletion in the p6^gag^ protein. (A) PBMCs were infected with fixed amounts of different HIV-1 subtype B (square) or CRF07_BC (circle) isolates and cultural supernatants were collected at days 4, 7, 11, 14, 18 and 21 post-infection. (B) The representative replicative curves of CRF07_BC and subtype B isolates deduced from Fig. 2A. (C) MT2 cells were infected with wild type (wt) or recombinant mutant virus with a 7 amino-acid deletion at the p6^gag^ (7d). Supernatants were collected at days 2, 4, 6, 8, 10, 12, and 14 post-infection. Viral replication was monitored through p24 antigen production. One-way analysis of variance (ANOVA) and Tukey's post hoc test were used to estimate the differences between subtypes, or between the recombinant viruses.

To confirm the findings mentioned above, we used co-culture methods to isolate CRF07_BC and subtype B strains from clinical specimens to compare their replication capacity. The results showed that the replication capacity of 9 CRF07_BC isolates was relatively lower than that of the 4 subtype B isolates (Fig. 2A). As shown in Fig. 2B, CRF07_BC isolates had significantly lower replication capacity than subtype B isolates.

### Both Subtype B and CRF07_BC Isolates were CCR5-tropic and Non-syncytia Inducing

Both genotypic and phenotypic assays were employed to compare the tropism between subtype B and CRF07_BC. In the genotypic assay, the C2-V5 regions of the *env* from 10 isolates were amplified using RT-PCR and the deduced amino acid sequences were aligned. Results indicated that the V3 loop sequences of all CRF07_BC isolates contained the typical GPGQ motif. Two of the three subtype B isolates had an amino acid deletion at position 25 of the V3 loop and all subtype B strains had the GPGR/K motif. Based on the 11/25 rule (presence of basic amino acid at positions 11 and 25 are X4 viruses) and the results from online tropism prediction (Geno2pheno and PSSM prediction programs), we concluded that all isolates were CCR5 tropic viruses. In the phenotypic prediction assay, results indicated that all isolates were R5 tropism and non-syncytium inducing (NSI) (Table 5).

**Table 5:** V3 amino acid sequences and predicted phenotypes of different HIV-1 isolates in Taiwan.

Isolate	Consensus V3 region amino acid sequences 11 1819 232425 CTRPNNNTRKSIHIGPGRAFYTTGEIIGDIRQAHC	Genetic subtype	Biotype	MT-2 assay	Predictions		
					Sequence^a^	Geno2pheno^b^	PSSM^c^
TW_D78	CTRPGNNTRK**S**IRIGPG**QT**FYA**TGD**IIGDIRQAHC	CRF07_BC	R5	NSI	R5	R5	R5
TW_D83	CTRPGNNTRK**S**IRIGPG**QT**FYA**TGD**IIGDIRQAHC	CRF07_BC	R5	NSI	R5	R5	R5
TW_D590	CTRPGNNTRK**S**IRIGPG**QT**FYA**TGD**IIGDIRQAHC	CRF07_BC	R5	NSI	R5	R5	R5
TW_D592	CTRPGNNTRK**S**IRIGPG**QT**FYA**TGD**IIGDIRQAHC	CRF07_BC	R5	NSI	R5	R5	R5
TW_D848	CTRPGNNTRK**S**IRIGPG**QT**FYA**TGE**IIGNIRQAHC	CRF07_BC	R5	NSI	R5	R5	R5
TW_D854	CTRPGNNTRK**S**IRIGPG**QT**FYA**TGD**IIGDIRQAHC	CRF07_BC	R5	NSI	R5	R5	R5
TW_D855	CTRPGNNTRK**S**IRIGPG**QT**FYA**TGD**IIGDIRQAHC	CRF07_BC	R5	NSI	R5	R5	R5
TW_D40	CTRPNNNTRR**S**IPIGPG**RA**FYT**SE-**IIGDIRKAHC	B	R5	NSI	R5	R5	R5
TW_D257	CTRPNNNTRK**S**ISMGPG**KA**FFA**TGD**IIGDIRAAYC	B	R5	NSI	R5	R5	R5
TW_D573	CTRPNNNTRK**S**IPIGPG**RA**FYT**TN-**IIGDIRKAHC	B	R5	NSI	R5	R5	R5

A dash indicated a deletion or lack of an insertion.

a The phenotype prediction based on 2 amino acid insertion/deletion between position 14 and 15, as well as variable amino acid positions (11, 18, 19, 23, 24 and 25) of V3 regions.

b Geno2pheno (http://coreceptor.bioinf.mpi-inf.mpg.de/index.php), false-positive rate of 0.01.

c Position-Specific Scoring Matrix (PSSM) (http://indra.mullins.microbiol.washington.edu/webpssm/).

### The Effects of a 7 Amino-Acid Deletion of p6^gag^ on Viral Life Cycle

Since co-receptor usage and SI properties of subtype B and CRF07_BC isolates are similar, we further studied the effect of a 7 amino-acid deletion of p6^gag^ on the viral life cycle. We generated HIV-1 NL4-3 recombinant virus containing a 7 amino-acid deletion in the p6^gag^ (7d virus). The replication capacity was analyzed by infecting MT-2 cells with wild-type (wt) and 7d viruses. Results showed that 7d virus replicated significantly slower than the wt virus (Fig. 2C).

**Figure 3:**
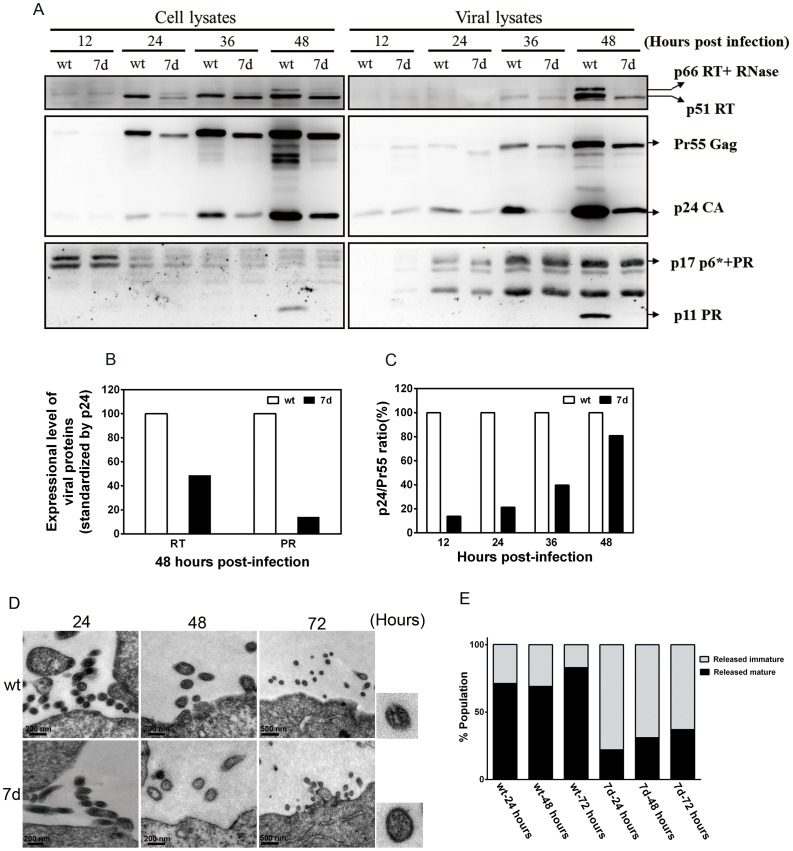
Characterization of the effects of a 7 amino-acid deletion in p6^gag^ to the HIV-1 proteins expression, release and maturation. MT2 cells were infected with wild type (wt) or deleted-type (7d) recombinant viruses. After 12, 24, 36 and 48 hours, supernatant was collected and pelleted by ultracentrifugation. (A) Western blot analysis of the cell lysates (left panel) and viral lysates (right panel) from cells infected with wt or 7d viruses. (B) The relative expression levels of PR and RT in the viral lysates of cells infected with wt or 7d virus. The total arbitrary densitometer units of PR and RT were standardized by p24 and normalized to those of wt in parallel experiments. The images were analyzed with Image J software. (C) The ratios of p24 vs. Pr55 (maturation index) in the viral lysates at different time points after infection were calculated. The total arbitrary densitometer units of each hours post infection were normalized to those of wt in parallel experiments. All results were representative of two independent experiments. (D) Electron microscopic (EM) examination of the viral particles of cells infected with wt or 7d recombinant viruses. MAGIC-5 cells were fixed and processed for transmission EM at different time points after they were infected with wt or 7d viruses. Scale bar indicates 200 nm. (E) Quantification of relative proportions of mature vs. immature virions released at different time points in the cells infected with wt or 7d viruses using EM. The method of virion quantification has been described previously [43].

To compare the efficiency of protease-mediated Gag processing and viral protein production in the wt and 7d viruses, we analyzed the reactive intensity of different protein bands in the viral lysates at 12, 24, 36 and 48 hours post-infection using WB assay. As shown in Fig. 3, compared to the wt virus, the level of viral proteins of 7d virus including p24, RT and PR appeared much lower in the cell lysates (24, 36 and 48 hours post-infection) and viral lysates (36 and 48 hours post-infection) (Fig. 3A). In addition, the relative expression levels of RT and PR of 7d virus in the viral lysates was significantly lower than those of the wt virus at 48 hours post-infection (Fig. 3B). Furthermore, we calculated the viral maturation index (the ratio of p24 to Pr55 in viral lysates) and found that 7d virus had significantly lower maturation index than the wt virus (Fig. 3C).

We also used electron microscopy to compare the morphogenesis between wt and 7d virions. We collected MAGIC-5 cells at different time points after infection with equal amounts of wt and 7d viruses and performed TEM. Results showed that more budding virions were observed in cells infected with wt virus than 7d virus (Fig. 3D). Besides, a higher percentage of immature virus particles released from cell membranes was found in cells infected with 7d virus at different time points (Fig. 3E).

**Figure 4:**
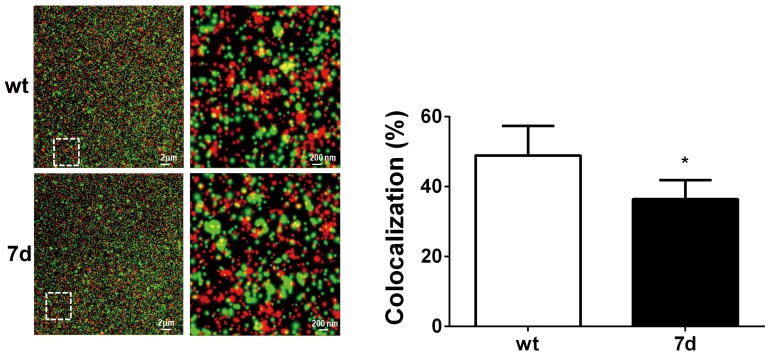
The interaction between Alix protein and wild type/mutant Gag. MAGIC-5 cells were infected with wt or 7d recombinant virus for 48 hours. The Alix and Gag proteins were analyze by TIRF-SR with rabbit anti-Alix polyclonal antibody and mouse anti-p24 monoclonal antibody. Red spots indicate Alix protein. Green spots indicate either wild type or 7d Gag protein. The proportion of co-localization of Alix and Gag protein was quantified using Volocity 3D Image Analysis Software.

Since the 7 amino-acid deletion overlaps with the Alix binding domain of p6^gag^, we conducted IFA staining with anti- p24^Gag^ and anti-Alix antibodies and analyzed their interaction using confocal microscopic exam with super-resolution program. The results showed that the co-localization coeffcicent of Gag and Alix was significantly lower in 7d virus than in wt virus (36.4% versus 48.88%, p<0.05) (Fig. 4).

## Discussion

Previously, several studies demonstrated that compared with patients infected with subtype B, patients infected with CRF01_AE had faster disease progression [28], [29]. While studies have compared the viral loads of patients with different subtypes including CRF01_AE, this study was the first to conduct a comparison of viral loads between patients infected with subtype B and CRF07_BC. We conducted a nested case control study using an HIV-1/AIDS patient cohort which we have followed up since 2010 to demonstrate that patients infected with CRF07_BC had significantly lower viral loads (about 58,000 copies/ml in average) than patients with subtype B infection (Table 4 and Fig. 1D). We also found that gender, HIV-1 subtype, CD4 cell count and ART were strongly associated with viral loads. Furthermore, a GEE model was performed to compare viral load differences in treatment naïve patients infected with subtype B and CRF07_BC at multiple time points followed up for more than two years and matched by age, gender and initial CD4 cell count. The results showed that when we matched the cases by initial CD4 cell count, the viral loads of patients infected with CRF07_BC were consistently lower than those patients with subtype B (Fig. 1D). The GEE model has been widely used to estimate the parameters of a generalized linear model with a possible unknown correlation between outcomes [30]. Since the focus of the GEE is on estimating the average response over the population rather than the regression parameters that would enable prediction of the effect of changing one or more covariates on a given individual, it can be used to determine the association between multiple time points of plasma viral loads or CD4 cell counts in patients and model the association between factors [31]–[33]. Previous studies demonstrated that male, older age and fast CD4 cell count depletion were significantly associated with higher viral loads [34], [35]. Variable disease progression rates among individuals infected with HIV-1 have been recognized, and different factors influencing clinical outcome have been demonstrated, including host genetic, immunological and virological aspects [36]. Examples of host factors include human leukocyte antigens (HLA) and chemokine co-receptor genotype, as well as the age of the individual at the time of infection. Virological characteristics have also been shown to affect pathogenicity, such as HIV-1 subtypes, chemokine co-receptor use, syncytium-forming properties, and viral fitness [1]–[3], [37]. Since plasma viral loads have been shown to be the best prognostic marker for disease progression, our data suggests that patients infected with CRF07_BC may have a much slower disease progression rate than patients infected with subtype B [38].

We performed growth kinetic analysis to compare subtype B and CRF07_BC primary isolates. CRF07_BC, as well as infectious recombinant viruses carrying a 7 amino-acid deletion in p6^gag^, showed significantly reduced replication capacity (Fig. 2). The data presented here indicated that CRF07_BC was associated with slower clinical progression in treatment naïve HIV-1/AIDS patients. Previously, several studies have demonstrated that the different rates of disease progression strongly correlated with different HIV-1 subtypes [1], [2]. The different co-receptor usage of HIV-1 can affect replication rate. In general, NSI virus (or R5 virus) replicates more slowly than SI virus (or X4 virus) [39]. Our data indicated that the all the Taiwanese CRF07_BC isolates tested were NSI and replicated in CCR5-expressing cells. Furthermore, we found that CRF07_BC strains showed patterns of moderate-level replication compared with subtype B in PBMCs. We suggest that the reason may be that the genome in CRF07_BC strains was mostly subtype C with only five regions from subtype B [8]. However, subtype C infected patients are extreme rare in Taiwan. It is difficult to compare the disease progression between CRF07_BC and subtype C infected patients in Taiwan. Previous studies showed that R5-tropic subtype C strains replicate more slowly in PBMCs than R5-tropic subtype B strains *in vitro* growth competition assays [40].

The 7 amino-acid deletion (residues 30 to 36) is quite unique to CRF07_BC. We previously reported that the 7 amino-acid deletion is unique among almost all the CRF07_BC strains isolated in Taiwan. One Taiwanese CRF07_BC strain even had a 11 amino-acid deletion in the p6^gag^ protein [8]. Subsequently, YM Shao's group sequenced 66 CRF07_BC strains from mainland China and found that the deletion was present in 25.8% of the cases [41]. As for whether other subtypes of CRFs have such deletion, according to our preliminary results using Los Alamos National Laboratory HIV-1 sequence database (http://hiv-web.lanl.gov) and data from YM Shao's paper, none of HIV-1 subtypes B, C CRF08_BC and other BC recombinants have this 7 amino-acid deletion [8]. In this study, we found that p6^gag^ containing a 7 amino-acid deletion showed moderate to severe defects in Gag processing and fewer viral enzymatic proteins in infected cell and virions (Fig. 3). HIV-1 protease-mediated gag processing and gag protein interaction with host cell proteins are very important for virus assembly, budding and maturation [42]. Tsg101 binding domain of HIV-1 p6^gag^ region (PTAP) was highly conserved in all the Taiwanese CRF07_BC strains, but they all have a 7 amino-acid deletion which overlaps with Alix binding domain (36-YPLASLRSL-44) at the residue 36Y [8]. A previous study demonstrated that Y36A mutation in p6^gag^ protein was critical for Alix interaction and virus budding [43]. HIV-1 release forms a viral bud and the connection between the membrane bud and plasma membrane needs to be disassociated [44]. In this study, we found that co-localization coefficient between Gag and Alix was significantly lower in 7d virus (Fig. 4). We therefore suggest that such deletions in p6^gag^ may affect its binding with Alix and subsequently affect virus release. In addition, Gag-Pol proteins are translated by -1 ribosomal frame shift during Gag translation [45]. Within the Gag-Pol, the p6^gag^ is truncated and replaced by a trans-frame domain referred to as p6^pol^ [46]. Deletions or mutations of p6^pol^ affect p6^pol^-PR disassociation and further impair PR activity [47]. Chiu et al performed a single – cycle infection assay and demonstrated that p6^pol^ deletion affected viral infectivity and reduced the p24/Pr55 protein ratio. [22]. A previous study demonstrated that truncation of p6^gag^ reduced the amount of viral enzymatic proteins in the virions [48]. Furthermore, our data showed that the 7 amino-acid deletion in p6^gag^ domain affected Gag processing efficiency and the amount of viral enzymatic proteins in HIV-1 virions. Further studies are needed to elucidate whether p6^pol^ containing a 7 amino-acid deletion affects the incorporation of gag-pol into virus particle or virus assembly in the nearby cytoplasmic membrane.

This is the first study that combines a longitudinal clinical follow-up study and a virological characterization of CRF07_BC infection. The Taiwanese CRF07_BC strains have about 97% nucleotide sequence homology with the prototypic CRF07_BC strains in China [8]. According to our previous phylogenetic analysis using the env gene, the Taiwanese CRF07_BC strains collected in 2004 formed at least two clusters with bootstrap value of 71 [8]. This phenomenon was reconfirmed by analysis of a larger number of CRF07_BC strains collected between 2005-2008, which showed a bootstrap value of 80 [10]. Therefore, there were more than 1 wave of CRF07_BC infection being transmitted to Taiwan and we believe that the findings in this study can also be applied to the CRF07_BC strains in mainland China.

In conclusion, our results suggest that patients infected with CRF07_BC have slower rate of disease progression and the deletion of 7 amino acids in its p6^gag^ region plays an important role in the assembly, budding and maturation processes of the viral life cycle.
